# Genome-wide characterization of the *PP2C* gene family in peanut (*Arachis hypogaea* L.) and the identification of candidate genes involved in salinity-stress response

**DOI:** 10.3389/fpls.2023.1093913

**Published:** 2023-01-27

**Authors:** Zhanwei Wu, Lu Luo, Yongshan Wan, Fengzhen Liu

**Affiliations:** ^1^ State Key Laboratory of Crop Biology, Shandong Agricultural University, Tai’an, China; ^2^ College of Agronomy, Shandong Agricultural University, Tai’an, China

**Keywords:** peanut, *PP2C* gene family, salt stress, genome-wide identification, gene expression analysis

## Abstract

Plant protein phosphatase 2C (PP2C) play important roles in response to salt stress by influencing metabolic processes, hormone levels, growth factors, etc. Members of the PP2C family have been identified in many plant species. However, they are rarely reported in peanut. In this study, 178 *PP2C* genes were identified in peanut, which were unevenly distributed across the 20 chromosomes, with segmental duplication in 78 gene pairs. AhPP2Cs could be divided into 10 clades (A-J) by phylogenetic analysis. *AhPP2C*s had experienced segmental duplications and strong purifying selection pressure. 22 miRNAs from 14 different families were identified, targeting 57 *AhPP2C* genes. Gene structures and motifs analysis exhibited *PP2C*s in subclades AI and AII had high structural and functional similarities. Phosphorylation sites of *AhPP2C45*/*59*/*134*/*150*/*35*/*121* were predicted in motifs 2 and 4, which located within the catalytic site at the C-terminus. We discovered multiple MYB binding factors and ABA response elements in the promoter regions of the six genes (*AhPP2C45*/*59*/*134*/*150*/*35*/*121*) by *cis*-elements analysis. GO and KEGG enrichment analysis confirmed *AhPP2C-A* genes in protein binding, signal transduction, protein modification process response to abiotic stimulus through environmental information processing. Based on RNA-Seq data of 22 peanut tissues, clade A *AhPP2C*s showed a varying degree of tissue specificity, of which, *AhPP2C35* and *AhPP2C121* specifically expressed in seeds, while *AhPP2C45*/*59*/*134*/*150* expressed in leaves and roots. qRT-PCR indicated that *AhPP2C45* and *AhPP2C134* displayed significantly up-regulated expression in response to salt stress. These results indicated that *AhPP2C45* and *AhPP2C134* could be candidate *PP2Cs* conferring salt tolerance. These results provide further insights into the peanut *PP2C* gene family and indicate *PP2Cs* potentially involved in the response to salt stress, which can now be further investigated in peanut breeding efforts to obtain cultivars with improved salt tolerance.

## 1 Introduction

Protein phosphorylation/dephosphorylation by protein kinases (PKs) and protein phosphatases (PPs) regulates targeted protein function in signal transduction cascades, leading to alterations in metabolism and/or gene expression in many biological processes, such as the development and stimulation of responses to the external environment (Lessard et al., 1997). PKs primarily phosphorylate serine (Ser), threonine (Thr) and tyrosine (Tyr), whereas PPs reverse this process (Li et al., 2014). PPs can be divided into three categories based on their substrate specificity: serine/threonine phosphatases, protein tyrosine phosphatases and dual specificity phosphatases (Kerk et al., 2008; Cao et al., 2016). Among them, protein tyrosine phosphatases can be further divided into phosphoprotein metallophosphatases and phosphoprotein phosphatases. Mn^2+^ or Mg^2+^-dependent protein phosphatases (PP2Cs) are important members of the phosphoprotein metallophosphatase family (Kerk et al., 2002).

PP2Cs are highly conserved in evolution and regulate stress signaling pathways in a targeted manner (Cao et al., 2016). Most PP2Cs in plants have a conserved catalytic region at the C-terminus, while the N-terminus is a poorly conserved region of varying lengths. The N-terminus is important for the definition of the PP2C member’s functionality, since this region can contain transmembrane regions and/or sequence motifs related with intracellular signaling, including those for interaction with kinases (Stone et al., 1994; Carrasco et al., 2003). It has been reported that some plant PP2Cs have significant roles in the plant response to environmental pressures, including salt, through their effects on metabolism, hormone levels and growth factors (Lim et al., 2015; Chu et al., 2021).

Relatively large PP2C gene families have been reported in several plant species, including soybean (103) (Chen et al., 2018), cotton (181) (Shazadee et al., 2019), *Arabidopsis* (76) (Kerk et al., 2002) and rice (132) (Singh et al., 2010). In *Arabidopsis*, PP2Cs (AtPP2Cs) are divided into clades A-J (Schweighofer et al., 2004). In clade A, ABA-INSENSITIVE 1 (ABI1) and ABA-INSENSITIVE 2 (ABI2) are important components of ABA signaling (Rodriguez et al., 1998; Kaliff et al., 2007). ABI1 and ABI2 act as negative regulators of ABA signaling, which play a pivotal role in the ABA-dependent salt stress response process. Under normal conditions, the ABA receptor (Pyrabatin Resistance 1-Like proteins; PYLs) is conformationally inactive and certain PP2Cs bind and dephosphorylate the sucrose non-fermenting-1-related protein kinases 2 (SnRK2) to inactivate ABA signaling. However, under osmotic stress ABA levels increase and their binding to the PYL induces conformational changes which enhance PYL-PP2C binding and inactivation of the PP2C. Consequently, the SnRK2 remains active and ABA-mediated stress signaling is initiated (reviewed in Gong et al., 2020; Zhang et al., 2022). Four members of the AtPP2Cs in clade B (APP2C1-4) are mitogen-activated protein kinase (MAPK) phosphatases that are negative regulators of phytohormones and defense responses (Ayatollahi et al., 2022). POLTERGEIST (AtPOL) and POLTERGEIST LIKE 1-5 (AtPLL1-5) of clade C are essential for cell maintenance and differentiation (Song et al., 2020). The functions of some AtPP2Cs in clades A, D, E, F and G have been reported, but the functions of most *Arabidopsis* PP2Cs remain unknown. The *PP2C* gene family in rice is divided into 11 clades, and most genes in clade A are triggered by various external abiotic stimuli, indicating that they play an important role in stress tolerance, particularly in salt stress (Xue et al., 2008).

Many *PP2C* gene family members have been reported to participate in plant salt stress regulation directly. (Leung and Giraudat et al., 1998; Kuhn et al., 2006; Liu et al., 2008). *AtPP2CG1* (*At2G33700*), a *PP2C* gene from *Arabidopsis*, is fully expressed under salt stress and positively regulates salt tolerance in an ABA-dependent manner (Liu et al., 2012). *At3G63320* and *At3G63340* actively respond to external stress stimuli and play a key role in the regulation of stomatal opening and closing, revealing that protein phosphorylation and dephosphorylation of signal regulators are significant for controlling stomatal pore size (Pang et al., 2020). Among *OsPP2Cs*, *OsPP2C53* (*Os05g51510*) and *OsPP2C51* (*Os05g49730*) are significantly expressed under 150mM NaCl stress (Xue et al., 2008). *OsPP2C08* (*Os01g46760*) is reported to be distinctly expressed in response to saline stress at the seedling stage compared to other *PP2Cs* (Sun et al., 2019). In rice, ubiquitination and degradation of OsPP2C09 (Os01g62760), a clade A PP2C, by abscisic acid-responsive RING Finger E3 Ligase (OsRF1) induced the salt tolerance of rice. (Kim et al., 2022).

Peanut (*Arachis hypogaea* L.) is an important oilseed and food crop worldwide. Cultivated peanut evolved from a cross between *Arachis duranensis* (A) and *Arachis ipaensis* (B) which subsequently underwent chromosome doubling to form the modern heterotetraploid (AABB genome, 2n=4x=40) with a total genome size of approximately 2.7 Gb (Bertioli et al., 2019). The main obstacles to peanut growth in semi-arid regions, where more than half of the global peanut production takes place, are drought and soil salinity (Banavath et al., 2018). There has been considerable advancement in the study of salt tolerance in peanut. Important examples include the stress-induced expression of *Arabidopsis* homeodomain-leucine zipper transcription factor (*AtHDG11*) to enhance salt tolerance, the identification of crucial intrinsic proteins that regulate salt stress in peanuts, the overexpression of the sodium/proton antiporter gene (*AtNHX1*) to improve salt tolerance, the vacuolar H^+^-pyrophosphatase gene (*AVP1*) (Asif et al., 2011; Banavath et al., 2018; Han et al., 2021). Compared to other classes of proteins, the role of AhPP2Cs in the peanut response to saline and other stresses are substantially less well understood. In order to advance the development of peanut salt-tolerant mechanisms, we identified the peanut *PP*2C gene family and selected genes associated with salt tolerance. In this study, we gained insights into the *AhPP2C* genes by using some computer analysis, such as characterization, genomic evolution, gene structure, motifs, *cis*-acting elements, catalytic sites and gene functional annotations, etc., and expression analysis in different tissues and under salt stress for research purposes. 178 *AhPP2C*s were identified in the *A. hypogea* genome. 18 members that clustered together with clade A of *Arabidopsis* were studied further. Of these, six *AhPP2C*s were clustered together with salt tolerant *PP2C* genes from rice and *Arabidopsis*. There were total 20 ABA response elements and 21 MYB binding sites in promoter region of the six genes (*AhPP2C45*/*59*/*134*/*150*/*35*/*121*). Moreover, their expression patterns under salt stress were analyzed from publicly available RNA-Seq data and qRT-PCR. *AhPP2C45* and *AhPP2C134* displayed significantly up-regulated expression under salt stress. They were considered as strong candidates for PP2Cs mediating salt stress signaling in peanut. These results provide theoretical support for biological functions of the peanut *PP2C* gene family under salt stress. Also, identification of *AhPP2C45*/*134* furnish new research threads for mechanism study of salt response differences between peanut germplasms, which play important roles in selection of salt tolerance germplasms and breeding.

## 2 Materials and methods

### 2.1 Identification of *PP2C* genes in cultivated peanut

Protein and gene sequences of *A. hypogaea* cv. Tifrunner and their annotated gene models were downloaded from PeanutBase^1^ (Version 1). Protein sequences were scanned using the HMMER v3 (Finn et al., 2011) using the hidden Markov model (HMM) for PP2Cs (PF00481) from the Pfam database^1^ (Finn et al., 2014). Proteins carrying the raw PP2C HMM with an E-value (≤1×10^-20^) were used to construct a peanut-specific hidden Markov model using hmmbuild from HMMER v3. The resultant peanut-specific HMM was used to search the protein sequences once again, and proteins that conformed to the criteria were submitted to NCBI-CDS^2^ (Conserved Domain Search) in order to select those containing the tPP2C domain, PF00481.

### 2.2 Phylogenetic analysis of *Arabidopsis* and peanut PP2Cs

Phylogenetic trees were constructed with MEGA11 software (Tamura et al., 2021) using the ClustalW algorithm (Thompson et al., 1994) for sequence alignment with the Maximum Likelihood (ML) method with 1000 bootstrap replicates and Pearson correction.

### 2.3 Chromosomal location and gene duplication analysis

The chromosomal locations of *AhPP2C*s were mapped with Map Gene 2 Chrom web v2^3^ (Chao et al., 2021). *AhPP2C* gene duplications were identified based on a sequence similarity of their CDS ≥75% and a sequence overlap of ≥75% of the longest sequence of the pair. Gene duplications were depicted using a circos diagram (Krzywinski et al., 2009). The Ka/Ks ratios of all *AhPP2C*s were predicted *via* simple Ka/Ks calculator in TBtools (Chen et al., 2020).

### 2.4 Phylogenetic analysis and physicochemical parameters of *AhPP2C-A*, salt tolerant *PP2C* genes from *Arabidopsis* and rice

*AhPP2Cs* in clade A were further clustered with *PP2C* genes associated with salt tolerance in *Arabidopsis* and rice. The physicochemical properties of *AhPP2C-A*, salt tolerant *PP2C* genes from *Arabidopsis* and rice, including amino acid length, molecular weight (MW), isoelectric point, were calculated using Expasy ProtPrarm tools^4^ (Wilkins et al., 1999).

### 2.5 Protein and gene structures, the prediction of the catalytic site and *Cis*-acting elements in *AhPP2Cs*

The gene structures of clade A *AhPP2Cs* were predicted and visualized using Gene Structure Display Server 2.0^5^ (Hu et al., 2015). Their conserved protein motifs were analyzed by MEME Suite Version 4.12.0 (Bailey and Elkan, 1994) using a maximum of 10 motifs with an a.a. length of 6-50. The other parameters were set as default. For *AhPP2C*s in subclades AI and AII, *cis*-acting elements were searched for in the 1500 bp region upstream of their start codons using PlantCARE^6^ (Lescot et al., 2002). Their protein secondary and tertiary structures were predicted using SOPMA^7^ (Geourjon and Deléage, 1994) and SwissModel^8^ (Waterhouse et al., 2018), respectively. The protein sequences were submitted to NCBI-Blast^9^, NetPhos3.1^10^ (Blom et al., 1999; Blom et al., 2004) and Phyre2^11^ (Kelley et al., 2015), for prediction of their catalytic sites.

### 2.6 Identification of miRNAs targeting *AhPP2C*s and gene function evaluation analysis

Through the psRNATarget website, the CDS of all *AhPP2C*s was used to predict miRNA target sites (Dai et al., 2018) with default. Figure of the interaction network among miRNAs and *AhPP2C*s were mapped by Cytoscape software (Shannon et al., 2003). Gene Ontology (GO) and Kyoto Encyclopedia of Genes and Genomes (KEGG) annotation evaluation was performed by submitting AhPP2C-A protein sequences to eggNOG-mapper^12^ (Cantalapiedra et al., 2021). GO and KEGG enrichment evaluations were undertaken using TBtools.

### 2.7 Plant material and treatment

In this study, the peanut cultivar Shanhua 11, which was bred and is maintained by our research group, was used as the plant material. Mature seeds were germinated on cotton wool soaked in distilled water within seed cultivation discs placed in darkness for three days at 26°C, after which, intact seedlings were transplanted to a hydroponic box and cultured with 1/5 Hoagland’s nutrient solution under a light/dark cycle of 16/8 h. Salt stress was applied to two-week-old seedlings by the adjustment of the nutrient solution to 200 mM NaCl. Leaves or roots from peanut seedlings were harvested after 72 h of salt or control treatments and were flash-frozen in liquid nitrogen before storage at -80°C until further use. Three biological replicates were used for analysis, each formed from materials obtained from five randomly chosen seedlings.

### 2.8 RNA extraction and qRT-PCR analysis

Total RNA was isolated with the Quick RNA Isolation Kit (Waryong, Beijing, China) and reverse transcribed into cDNA using Advantage RT-for-PCR Kit (TaKaRa, Dalian, China) following the manufacturer’s instructions. The specific primers were designed using Beacon Designer 7.9. SYBR Green real-time PCR was performed according to the guidelines of the PCR machine. The relative expression levels were calculated by the 2^–ΔΔCt^ method (Livak and Schmittgen, 2001). Statistical differences were determined by T-test (**P<0.01, *P<0.05).

### 2.9 The analysis of *AhPP2C* gene expression in peanut tissues and seedlings under salt stress

The tissue specificity of *AhPP2Cs* was determined from RNA-Seq data obtained for 22 different tissues at different developmental stages during peanut growth (Clevenger et al., 2016) obtained from the peanut database. The RNA-Seq data from salt-stressed peanut seedlings (Luo et al., 2021) was obtained from the public repository of NCBI^13^ under the biological project accession number, PRJNA560660. The FPKM values of *AhPP2C-A*, including subclades AI and AII and other *AhPP2Cs* were extracted from the data set. The heatmap of was generated by TBtools after log2- transformation of FPKM values.

## 3 Results

### 3.1 Identification and phylogenetic analysis of the peanut *PP2C* gene family

**Figure 1:**
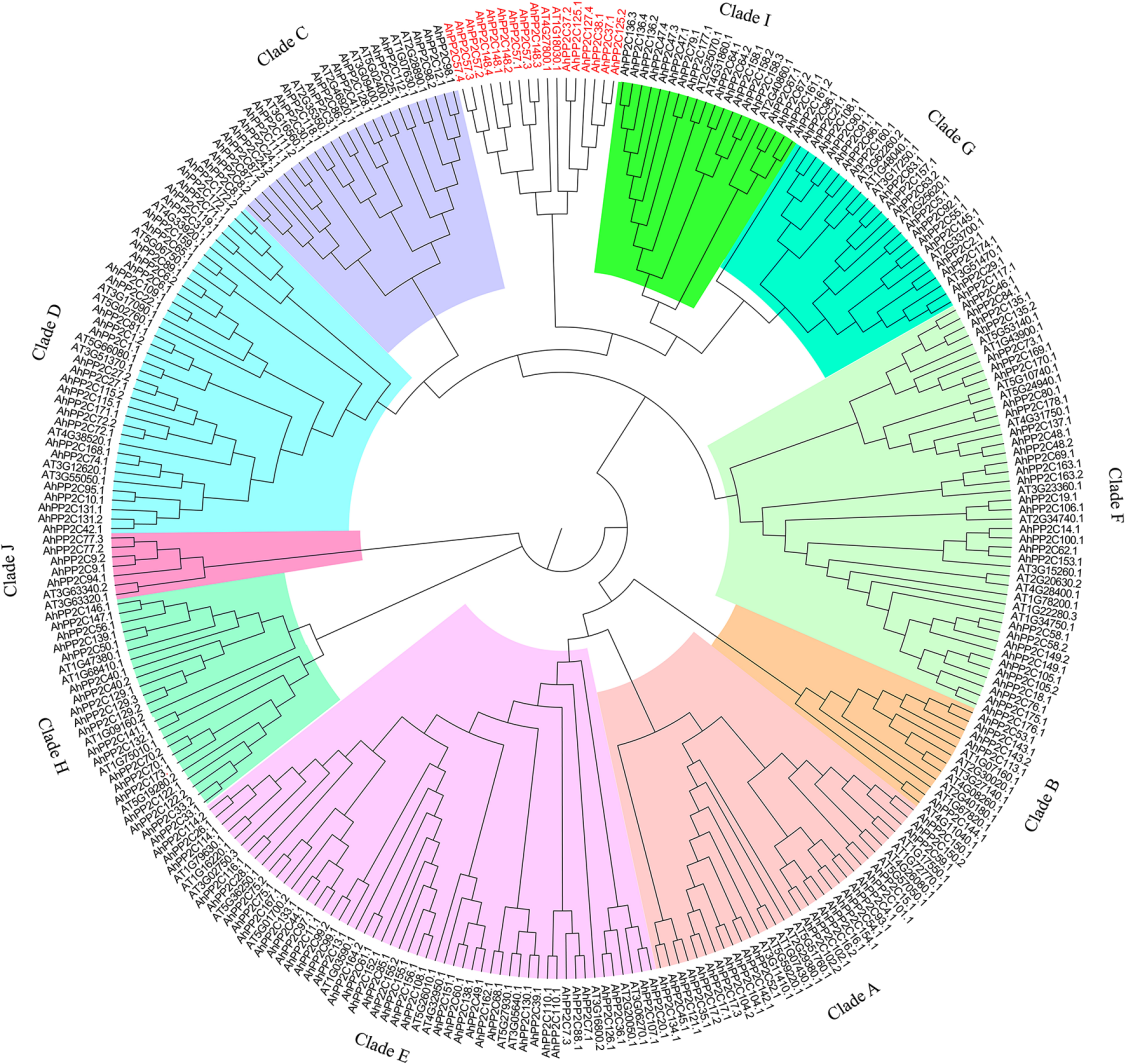
A Phylogenetic tree of PP2Cs from peanut and *Arabidopsis*. PP2Cs in different clades are labeled with different colors. 6 *AhPP2C* genes in red were not classified.

A total of 183 PP2C-coding candidate genes were identified *via* HMM searching in peanut (*A. hypogaea* L.). After screening for the presence of the typical PP2C domain (PF00481), 178 genes were retained. A phylogenetic tree was created after multiple sequence alignment of the 178 *AhPP2C*s 76 *Arabidopsis PP2C* genes. The 178 *AhPP2C*s were divided into the 10 clades, A-J, as reported in other plants before, while 6 *AhPP2C*s did not belong to any reported *PP2C*s clades (**Figure 1** and **Supplementary Table 1**). Of these, clade E was the largest, containing 34 *AhPP2Cs*, while clade B was the smallest, including 4 *AhPP2Cs*. The cladistics analysis indicated that the *PP2C*s of peanut and *Arabidopsis* were similarly distributed between the clades, indicating the maintenance of the same types of functional diversity in *PP2C*s following their speciation.

### 3.2 Chromosomal distribution and gene duplication analysis of *AhPP2Cs*

**Figure 2:**
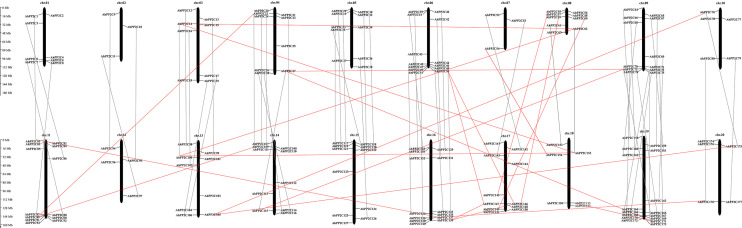
Chromosomal distribution of *AhPP2C*s. Orthologous genes were linked by gray lines. Paralogous genes were linked by red lines.

**Figure 3:**
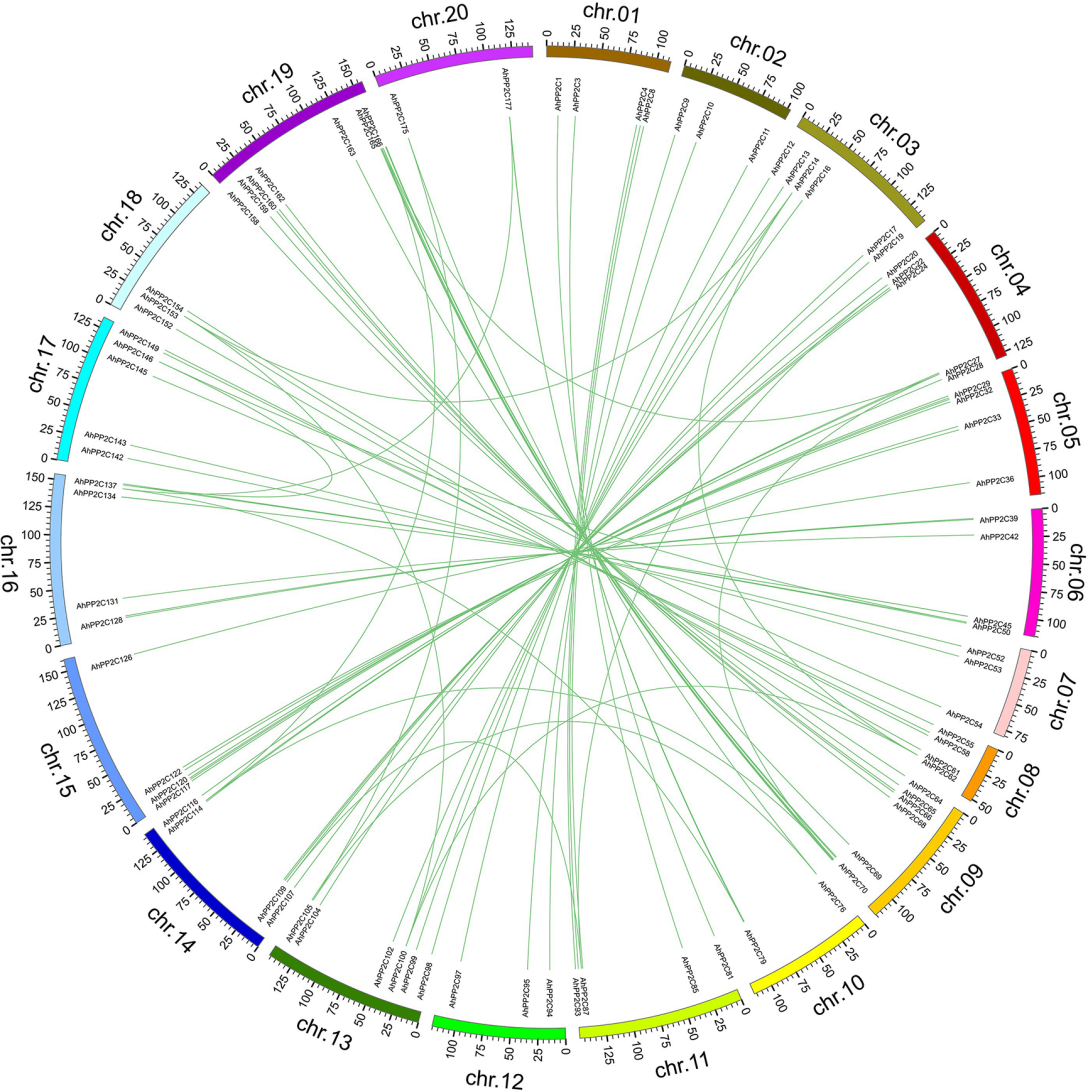
Gene duplication analysis of *AhPP2C*s. Chromosomes are drawn in different colors. The scale provided represents the chromosome size (Mbp). Duplicated *AhPP2C*s are linked by green lines.

As shown in **Figure 2**, all 178 *AhPP2C*s are unevenly distributed on 20 chromosomes, with no more than five genes on chromosomes 2, 7, 10 and 12, while the others are greater than five, with the greatest number of genes on chromosome 20. Most *AhPP2Cs* were concentrated near the ends of each chromosome, which was similar to the chromosomal distribution of PP2Cs reported for other plant species (Shazadee et al., 2019; Yu et al., 2019). A gene duplication analysis based on sequence similarity of *AhPP2Cs* indicated 78 genes pairs and found that there was segmental duplication in 78 genes pairs (**Figure 3** and **Supplementary Table 2**). As expected, the paired *AhPP2Cs* clustered together in the phylogenetic tree. However, their distribution positions on 20 chromosomes were similar. Thus, to understand the mode of selection of the *AhPP2C*s, the Ka, Ks, and Ka/Ks ratio was revealed for all gene pairs (**Supplementary Table 2**). The dataset unveiled that all duplicated *AhPP2C* gene pairs had a Ka/Ks ratio of <1, except for *AhPP2C54*/*154*, suggesting that *AhPP2C*s in these groups underwent purifying selective pressure. The Ka/Ks value of two gene pairs (*AhPP2C42*/*131*, *AhPP2C81*/*137*) was zero, indicating strong purifying selection.

### 3.3 Investigation of putative miRNAs targeting *AhPP2C* genes

**Figure 4:**
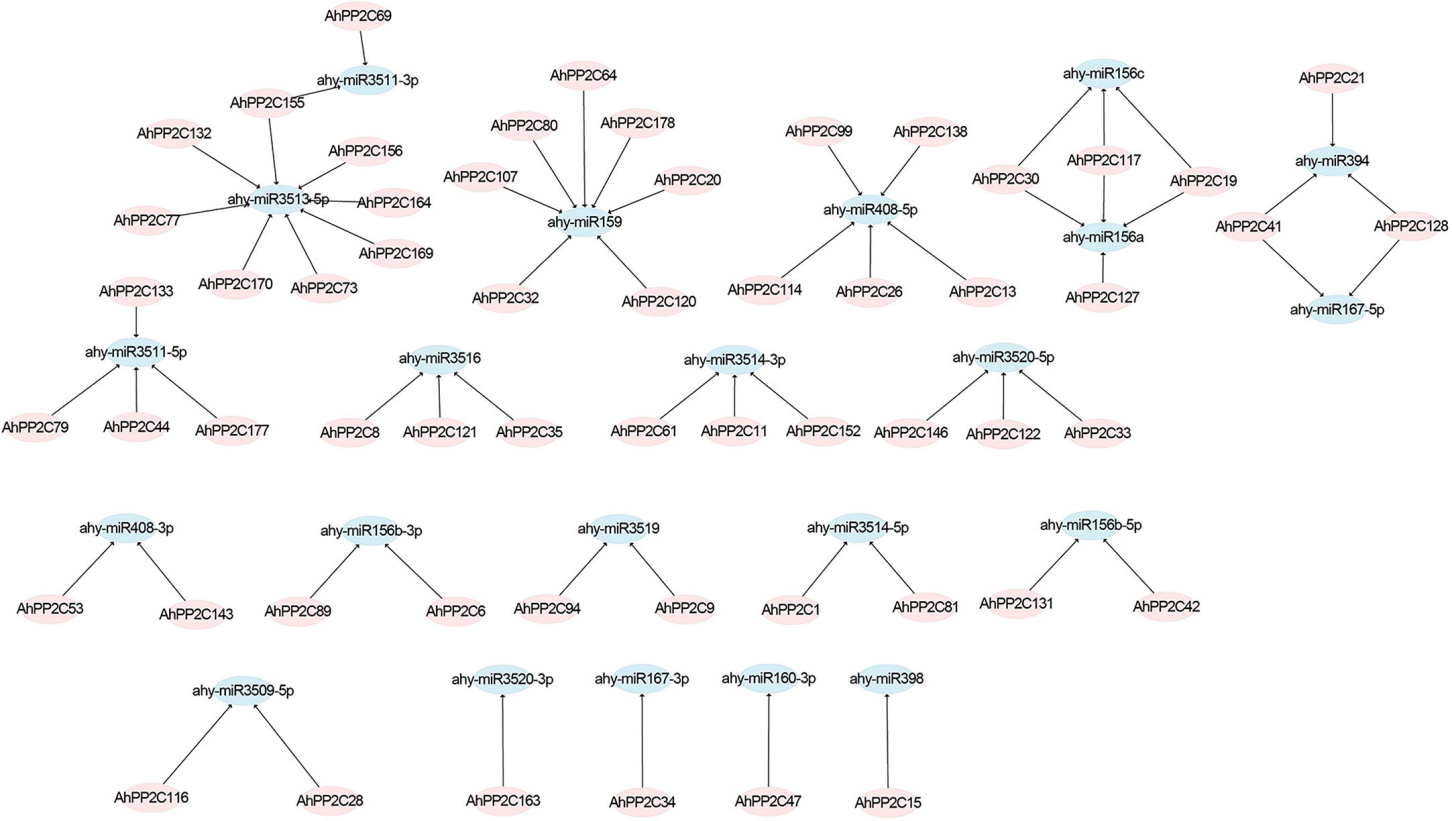
Prediction of putative miRNAs targeting *AhPP2C*s. The pink shapes correspond to *AhPP2C*s, and blue shapes indicate predicted miRNAs.

To better investigate how the *AhPP2C* genes were regulated by miRNAs during translation, we identified 22 miRNAs targeting 57 genes (**Figure 4** and **Supplementary Table 3**). These miRNAs belonged to 14 different families. The result showed that ahy-miR3513-5p targeted the most number (8) of genes, followed by ahy-miR159 that targeted seven genes. Two miRNAs, including ahy-miR156a and ahy-miR3511-5p, targeted four genes, followed by ahy-miR156c, ahy-miR394, ahy-miR3516, ahy-miR3514-3p and ahy-miR3520-5p that targeted three genes, while ahy-miR408-3p, ahy-miR156b-3p, ahy-miR3519, ahy-miR3514-5p, ahy-miR156b-5p and ahy-miR3509-5p targeted two different genes. Only four miRNAs, including ahy-miR3520-3p, ahy-miR167-3p, ahy-miR160-3p and ahy-miR398 targeted one gene, *AhPP2C163*, *AhPP2C34*, *AhPP2C47* and *AhPP2C15* respectively. In *AhPP2C-A*, only *AhPP2C15*, *AhPP2C35* and *AhPP2C121* were targeted by miRNAs. Some genes like *AhPP2C30*, *AhPP2C117 AhPP2C19*, *AhPP2C41*, *AhPP2C128* and *AhPP2C155* were found to be targeted by more than one miRNA.

### 3.4 Phylogenetic analysis and physicochemical parameters of *AhPP2C-A*

**Figure 5:**
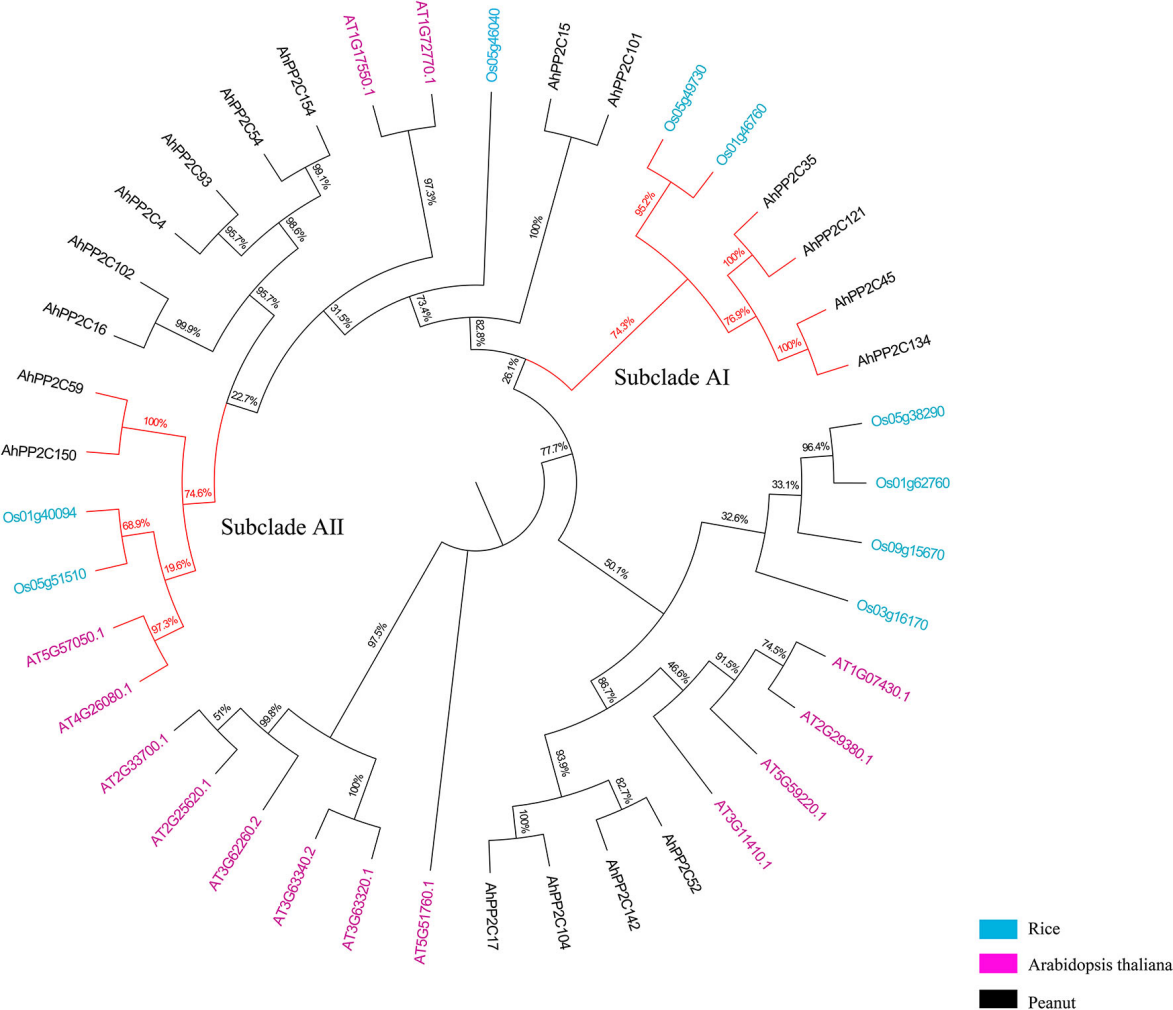
Phylogenetic tree of clade A *AhPP2Cs*, with salt tolerant genes from *Arabidopsis* and rice. The branches of subclades AI and AII are indicated in red.

Previous studies have demonstrated that clade A *PP2C* genes has been identified to be associated with salt tolerance in rice and *Arabidopsis* (Schweighofer et al., 2004; Xue et al., 2008). In peanut, eighteen *PP2C* genes belong to clade A. To further investigate their biological functions, *AhPP2C-A* were further compared with the reported salt response *PP2C* genes in rice and *Arabidopsis*. As shown in **Figure 5**, the latter were clustered together with closely related *AhPP2C*s in subclades AI and AII. The bootstrap values of these two branches were 74.3% and 74.6%, respectively, indicating a high degree of confidence, suggesting that *AhPP2Cs* in these subclades may be functionally associated with salt tolerance. Their identification regresses to finding peanut sequences with the high homology with known *PP2C* genes associated with salt tolerance. The physicochemical properties of subclades AI and AII are shown in **Table 1**, which indicates that all members of each subclade shared similar physiochemical properties.

**Table 1:** Physicochemical parameter of PP2Cs in Subclades AI and AII.

Subclade	Name	Numbers of Amino Acid/aa	MW/kDa	Theoretical pI
AI	AhPP2C35	346	36.9	5.22
	AhPP2C121	346	37.2	5.24
	AhPP2C45	380	41.5	6.1
	AhPP2C134	380	41.5	5.78
	Os05g49730	381	40.3	7.64
	Os01g46760	403	43.0	5.57
AII	Os01g40094	356	36.4	4.68
	Os05g51510	445	46.7	4.90
	AT5G57050.1	423	46.3	5.93
	AT4G26080.1	434	47.5	5.81
	AhPP2C150	552	59.4	4.73
	AhPP2C59	553	59.5	4.74

### 3.5 Gene structures and motifs analysis of *PP2C*s in subclades AI and AII

**Figure 6:**
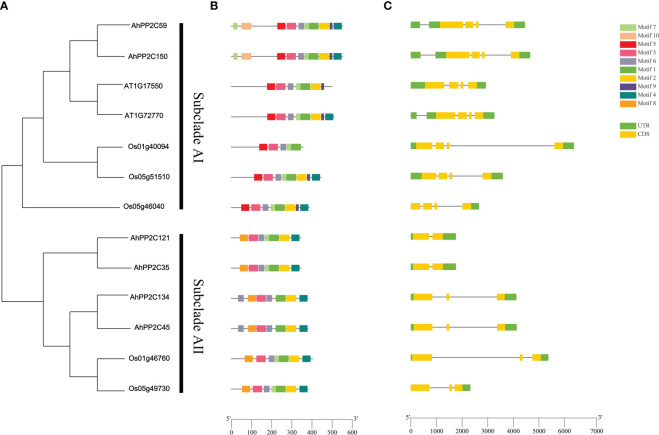
Phylogenetic relationship, gene structures and motifs analysis of PP2Cs in subclades AI and AII. **(A)** Phylogenetic tree of PP2Cs **(B)** Motifs analysis of PP2Cs. Colored boxes indicate the different conserved motifs as indicated in the scheme to the right of the figure. **(C)** Exon-Intron Structure of *PP2C*s, where exons are shown as yellow boxes. Where present, 5’ and 3’ UTRs are depicted as green boxes.

To further analyze the potential roles of *AhPP2C*s in subclades AI and AII, their phylogeny was analyzed together with their gene structures and motifs (**Figure 6**). In subclade AI, all *PP2Cs*, including the salt tolerant genes from *Arabidopsis* and rice in the same branch of the phylogenetic tree were similar in structures and contained 4 exons. In subclade AII, *AhPP2C134* and *45*, and two rice *PP2C*s involved in the response to salt stress contained 3 exons, while the remaining two *AhPP2Cs* contained only 2 exons.

A total of 10 motifs was identified using MEME Suite. Motifs 5, 9 and 10 were exclusive to subclade AI, motif 8 was exclusive to subclade AII, while motifs 1, 2, 3, 4, 6 and 7 were present in both subclades. After alignment of protein sequences, conserved domain sequences and motifs, it was found motifs 2 and 4 are located in the catalytic site at the C-terminus (Bork et al., 1996). These sites were highly conserved across the PP2Cs of subclades AI and AII, except Os1g40094 (**Figure 6** and **Supplementary Figure 1**). Motif 10 was located at the N-terminal, but it appeared to be highly conserved, because it was found to exist on fewer number of sequences (**Supplementary Table 4**). Motif 5 and motif 9 were only present in subclade AI. This may account for the functional differences between the two subclades. AhPP2C45/134 in subclade AII had a greater number of motif 6, where it may confer unique functions to these PP2Cs, which will need be investigated further.

### 3.6 Protein structure and catalytic site prediction of *AhPP2Cs* in subclades AI and AII

**Figure 7:**
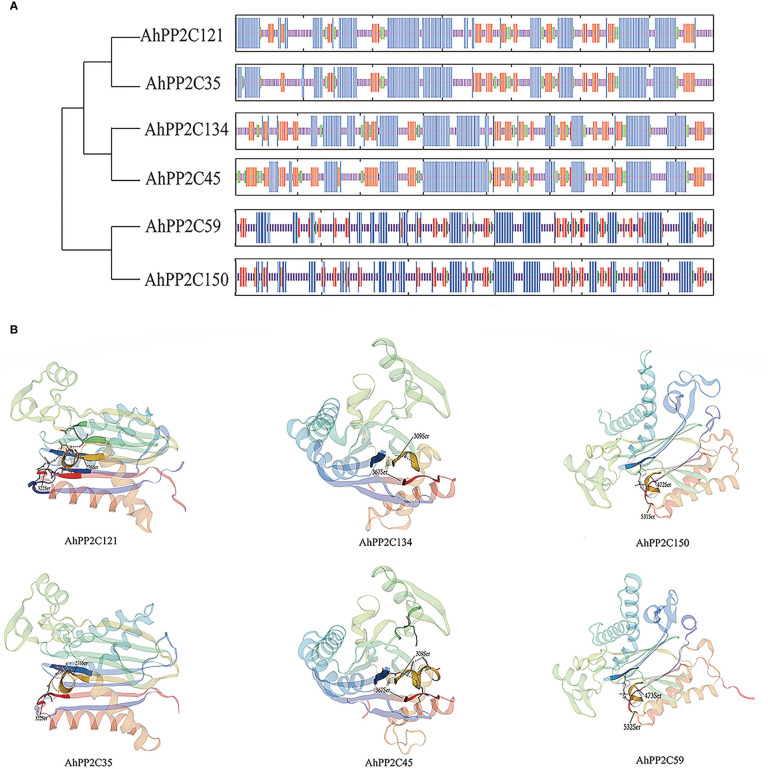
Protein structures and catalytic site prediction of *AhPP2C*s in subclades AI and AII. **(A)** protein secondary structures of *AhPP2C*s. α-helices were in blue; irregular curls were in purple; β-sheets were in red; β-turns were in green. **(B)** protein tertiary structures and catalytic sites of *AhPP2C*s in subclades AI and AII.

The secondary structures of *AhPP2Cs* in Subclades AI and AII consisted of 31.16%-43.64% α-helices, 30.26%-46.11% irregular curls, 15.91%-23.68% β-sheets and 5.25%-9.21% β-turns. Analysis of the secondary structures of orthologous gene pairs *AhPP2C150*/*AhPP2C59*, *AhPP2C134*/*AhPP2C45* and *AhPP2C121*/*AhPP2C35* showed they were very similar. The tertiary structures were also highly similar (**Figure 7**). Moreover, their catalytic structural domains all consist of a central β-sandwich that binds Mn^2+^/Mg^2+^ and is surrounded by α-helices (Das et al., 1996; Shi, 2009). The phosphorylation sites, which can be found in motifs 4 and 2 within their predicted catalytic sites were also found to be similar: S362 and S309 for AhPP2C134/AhPP2C45, S322 and S276 for AhPP2C35/AhPP2C121, S532 and S473 for AhPP2C59 and S531 and S472 for AhPP2C150.

### 3.7 Gene ontology and Kyoto Encyclopedia of genes and genomics enrichment analysis of *AhPP2C-A* genes

To further understand the role of *AhPP2C-A* genes at the molecular level, we performed GO and KEGG enrichment analysis (**Supplementary Figure 2**). The GO enrichment analysis can be divided into three main categories: biological processes (BP), molecular functions (MF) and cellular components (CC). For instance, in MF class, the most enriched term was protein binding (GO:0005515), followed by binding (GO:0005488) and hydrolase activity (GO:0016787). In CC class, the highly enriched terms were cytosol (GO:0005829) and nucleus (GO:0005634). Whereas in BP class, the highly enriched terms were cell communication (GO:0007154), signal transduction (GO:0007165), regulation of molecular function (GO:0065009), response to endogenous stimulus (GO:0009719), response to abiotic stimulus (GO:0009628), etc. In addition, KEGG pathway enrichment study identified four pathways involved in the different functions of the *AhPP2C-A* genes. The highly enriched pathways include signal transduction (B09132), plant hormone signal transduction (04075), environmental information processing (A09130), MAPK signaling pathway – plant (04016) (**Supplementary Table 5**). In brief, it could be concluded that *AhPP2C-A* genes related to abiotic stresses response, protein modification process and plant hormone signaling.

### 3.8 *Cis*-acting elements analysis of *AhPP2C*s in subclades AI and AII

In higher plants, PP2C is involved in many stress resistance responses and is induced by a variety of abiotic stresses. *Cis*-acting elements were therefore searched in the 1500 bp sequences upstream of the start codons of peanut *PP2Cs* of subclades AI and AII (**Table 2**). There were obvious differences in the types and numbers of *cis*-acting elements among different *AhPP2C*s. The results showed that many phytohormones and stress-related response elements were identified, including ABRE/ABRE4/ABRE3a, Aux RR-core/TGA-element, TCA-element, MYB and CGTCA-motif/TGACG-motifs. *AhPP2C*s in subclades AI and AII all contained MYB binding factors, which are essential for regulating how plants respond to abiotic stress, particularly in response to salt stress. *AhPP2C150*, *35*, *134 and 45* contained *cis*-acting elements involved in the regulation of the ABA response (ABRE/ABRE4/ABRE3a), while *AhPP2C121* and *59* did not. *AhPP2C45* contained the maximum number of *cis*-elements (21), while *AhPP2C35* contained the minimum number of *cis*-elements (4). The differences in number and kind of elements may lead to functional difference of *AhPP2C*s.

**Table 2:** The Distribution of *Cis*-Acting Elements in *AhPP2C*s of Subclades AI and AII.

Gene name	Numbers of *cis*-acting element								
	A	B	C	D	E	F	G	H	I
*AhPP2C150*	2	3	—	3	—	—	5	2	2
*AhPP2C121*	—	—	1	—	—	1	3	—	1
*AhPP2C35*	1	1	1	—	—	—	1	—	—
*AhPP2C134*	7	—	2	—	2	—	6	2	—
*AhPP2C59*	—	—	—	1	—	—	2	—	2
*AhPP2C45*	10	—	2	—	2	—	4	2	1

Key: A, ABRE/ABRE4/ABRE3a; B, Aux RR-core/TGA-element; C, ERE; D, TCA-element; E, MBS; F, LTR; G, MYB binding factor; H, CGTCA-motif/TGACG-motif; I, WUN-motif; —, no response element.

### 3.9 Expression profiling of clade A *AhPP2C*s in different tissues

**Figure 8:**
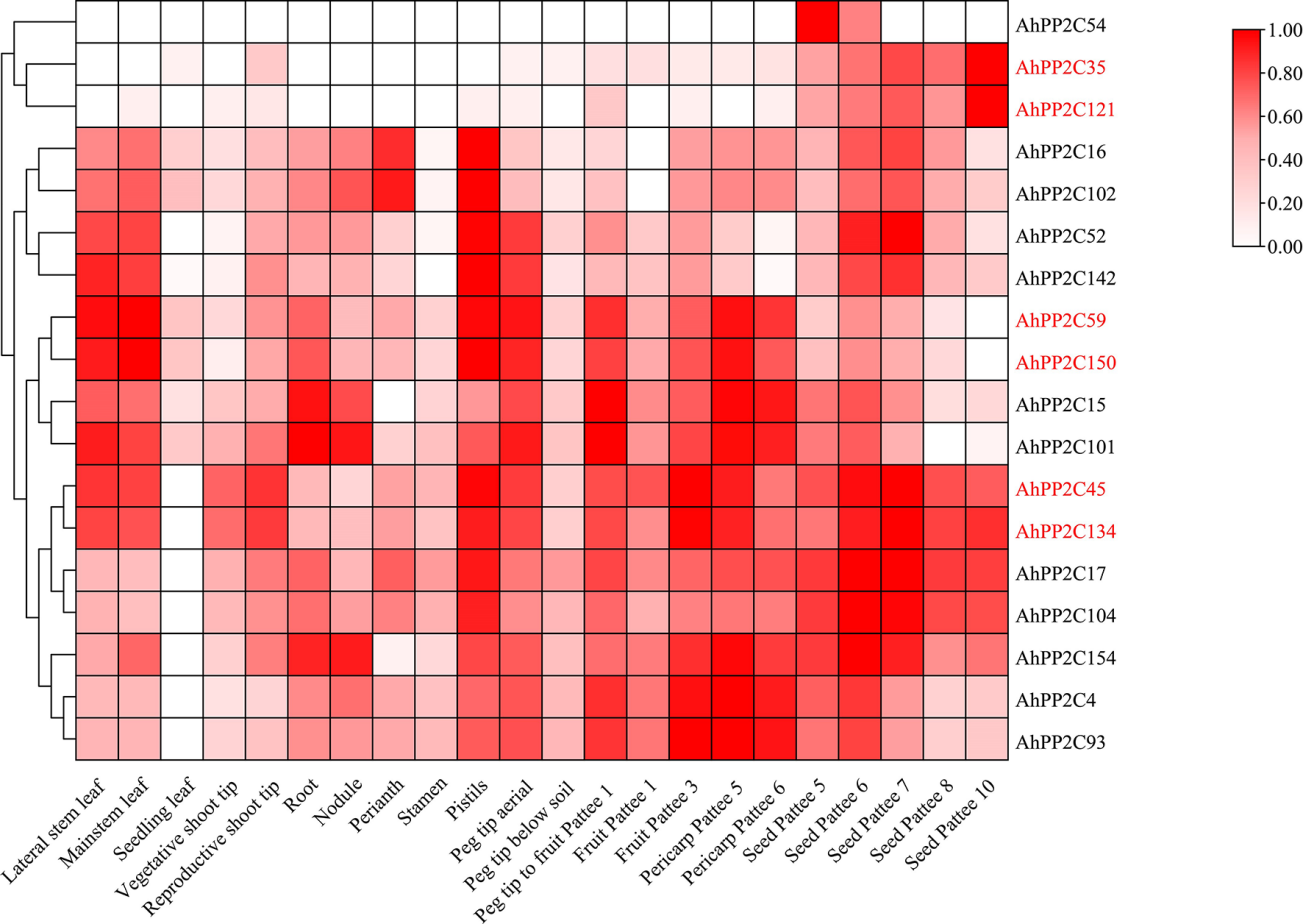
Expression pattern analysis of clade A *AhPP2Cs*. The heat map of log2-transformed averaged FPKM data was mapped using the intensity scale shown on the right. The visualization and clustering utilized TBtools. The *AhPP2Cs* of subclades AI and AII are labelled in red.

Based on the transcriptome data of 22 peanut tissues (Clevenger et al., 2016), the expression patterns of clade A *AhPP2Cs* were extracted and shown in **Figure 8** and **Supplementary Table 6**. Clade A *AhPP2C*s diverse with differing levels of tissue specificity. *AhPP2C17*, *59*, *150*, *134*, *45*, *15 and 101* expressed at relatively high levels in several of the 22 different tissues. In contrast, the overall expression levels of *AhPP2C54, 35* and *121* were lower, and mainly detected in seeds tissues. The remaining genes (7) were moderately expressed in all tissues. Considering *AhPP2C*s of subclades AI and AII, *AhPP2C121* and *35* were only expressed in seeds, while the other four genes were also expressed in leaves and roots. The above results indicated that clade A *AhPP2C*s exhibited different expression patterns.

### 3.10 The salt stress effects on the expression of *AhPP2C*s in subclades AI and AII

**Figure 9:**
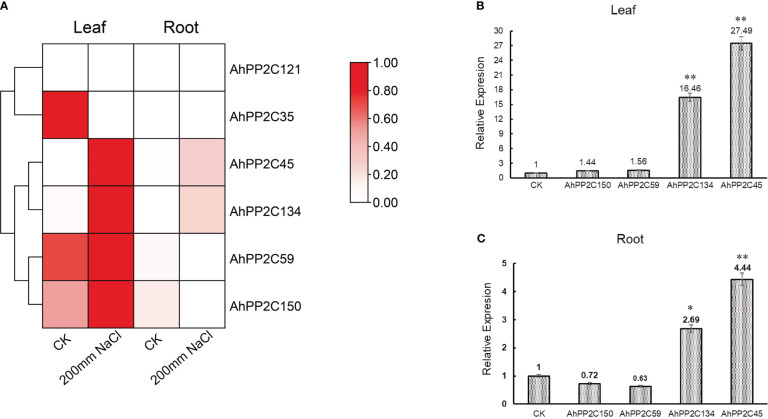
Expression profiles under salt stress. **(A)** Relative expression pattern of *AhPP2C*s in subclades AI and AII in response to salt stress. **(B, C)** qRT-PCR analysis of *AhPP2C*s *45*, *59*, *134* and *150* under salt stress in leaves **(B)** and roots **(C)**. “*” indicated that the genes were significant differences compared with the untreated control (**P<0.01, *P<0.05).

An initial assay of the expression of subclade AI and AII *AhPP2Cs* in seedlings under salt stress used publicly available RNA-Seq data from leaves and roots. The heatmap of relative expression is shown in **Figure 9A**. Under salt stress, in leaves, *AhPP2C45*, *134*, *59* and *150* were up-regulated compared with the control, but this was only significant in *AhPP2C45* and *134*. In roots, *AhPP2C45* and *AhPP2C134* were also up-regulated, while *AhPP2C59* and *AhPP2C150* were down-regulated relative to the control. The expression of *AhPP2C121* was not detected in roots or leaves with or without salt stress.

To validate the results for the four up-regulated genes under salt stress one step further, the relative expression levels of these *AhPP2Cs* (*45, 134, 59 and 150*) in leaves and roots were analyzed by qRT-PCR (**Figures 9B, C**). After 72 h of salt treatment (200 mM NaCl), the relative expression levels of *AhPP2C45* and *134* were up-regulated significantly in both leaves and roots. These results indicated that AhPP2C45 and 134 may play important roles in peanut salt stress response.

## 4 Discussion

Salt stress is the second worst abiotic factor affecting global agricultural productivity, disrupting many physiological, biochemical and molecular processes in plants. Salinity affects plant growth, development and productivity (Raza et al., 2022). Salt stress also has a serious impact on peanut production (Aravind et al., 2022). PP2Cs are widespread and highly conserved in prokaryotes and eukaryotes (Yang et al., 2018). Several studies of plant PP2Cs have indicated that of a subfamily of PP2Cs (clade A) have regulatory roles in stress responses through dephosphorylating the substrate proteins, especially in ABA-dependent responses, such as the drought and salinity response (Xue et al., 2008; Cui et al., 2013; Singh et al., 2015; Krzywińska et al., 2016). However, less information is available for *PP2C*s in the economically important crop plant peanut, whose productivity is considerably affected by salinity stresses. This research therefore aimed to provide a systematic analysis of *AhPP2C*s and to identify candidate *AhPP2C*s participate in salt stress response.

In this study, we identified 178 *AhPP2C*s that showed a phylogenetic clustering similar to that of *Arabidopsis*, including a distinct clade A, which is considered to contain a subfamily of *PP2C*s with roles in ABA-mediated salt stress responses (Zhang et al., 2013; Singh et al., 2015; He et al., 2019). Numbers of *PP2C* genes differ a lot among species (Kerk et al., 2002; Chen et al., 2018; Shazadee et al., 2019). Deviations in the number of *PP2C* members from different plant species may be attributed to gene duplication events, including tandem duplication and segmental duplication, which play an important role in expanding *PP2C*s members. Duplications of *PP2C* genes have also been found in some plant species (Yang et al., 2018; Zhang et al., 2022). Our results confirmed that *AhPP2C*s had underwent segmental duplication strong purifying selective pressure (**Figure 3** and **Supplementary Table 2**). Previous phylogenetic tree of *Arabidopsis* showed that there were 6 *AtPP2C*s could not clustered to any clades (Schweighofer et al., 2004; Bhaskara et al., 2019). In this work, we found 6 *AhPP2C*s clustered with 2 of the 6 *AtPP2C*s, and we speculated that this may be universal phenomena in plant *PP2C* gene family. Some AhPP2Cs of clade A clustered together with PP2Cs known to confer salt tolerance in *Arabidopsis* and rice (**Figure 5**). The AhPP2C35, 121, 45 and 134 of subclade AI were found to be closely clustered with the OsPP2Cs Os05g49730 (OsPP2C51) and Os01g46760 (OsPP2C08). *OsPP2C08* (*Os01g46760*) was identified as a potential candidate gene conferring improved salt tolerance through the integration of genome-wide polymorphism information with QTL mapping and expression profiling data (Subudhi et al., 2020). The heterogeneous expression of *OsPP2C51* (*Os05g49730*) in *Arabidopsis* revealed that it could positively regulate ABA-induced rice seed germination under conditions of salt stress (Bhatnagar et al., 2017).

Similarly, the AhPP2C59 and 150 of subclade AII were closely clustered with two rice Os01g40094 (OsPP2C06) and Os05g51510 (OsPP2C53) and two *Arabidopsis* PP2Cs (AT5G57050.1 and AT4G26080.1). ABI2 (AT5G57050.1) as a SOS2-interacting protein can affect salt stress response in *Arabidopsis* (Ohta et al., 2003). Mutations ABI1 (AT4G26080.1), affecting ABA-perception in *Arabidopsis*, can reduce the accumulation of both AtP5CS mRNAs during salt-stress and result in higher proline accumulation. (Strizhov et al., 1997). OsPP2C53 (Os05g51510) negatively regulates OsSLAC1 in stomatal closure and transgenic rice overexpressing *OsPP2C53* showed significantly higher water loss than control (Min et al., 2019). Microarray analysis of transgenic rices overexpressing *OsNAP* in high salinity, drought and low temperature revealed that a stress-related gene *OsPP2C06* (*Os01g40094*) was up-regulated (Chen et al., 2014). Salt tolerant *PP2C* genes from *Arabidopsis* and rice are located in both subclades AI and AII, then this functionality occurs in two phylogenetically distinct subclades. This could, for example, be because function of salt tolerant *PP2C* genes can be achieved in mechanistically different ways and these evolved independently, or these genes differ in other aspects of their sequence (domains/motifs) that have more weight in their phylogenetic placing. In addition, the peanut *AhPP2Cs* that closely clustered with *PP2Cs* conferring salt tolerance also shared similar motifs and gene structures with their *Arabidopsis* and rice counterparts (**Figure 6**), indicating that they may also share similar functionalities.

Additionally, we found nine *cis*-elements, two of them, ABRE/ABRE4/ABRE3a and MYB binding factor are related to salt tolerance. ABRE/ABRE4/ABRE3a has been discovered in previous studies of *PP2C*s in stress (Xue et al., 2008; Chen et al., 2018), however there is less available information regarding MYB binding factors in this process. Furthermore, *AhPP2C*s gene functions were further predicted by GO enrichment analysis, which supported the role of these genes in dephosphorylating the substrate proteins and salt stress response. It is therefore of interest that after our analysis of the expression profiles of *AhPP2C*s under salt stress, we observed the *PP2C* genes with ABRE/ABRE4/ABRE3a and MYB binding factors showed greater relative changes in expression. The genes (*AhPP2C45*/*134*) contained both *cis*-elements and having the maximum number of both *cis*-elements. The greater number of salt tolerance-related cis-elements *AhPP2C*s have, the greater expression levels under salt stress. The number of salt tolerance-related *cis*-elements may therefore be correlated with either high or low gene expression in response to salt stress.

The potential phosphorylation sites and protein structures of the AhPP2Cs were also examined. The conserved catalytic core domain of PP2Cs contains a central β-sandwich with a pair of α-helices on either side of each β-sheet (Das et al., 1996). Within the conserved catalytic structural domain, the PP2Cs contain three characteristic sequences: DG××G, DG, and G××DN (where x is any amino acid) (Shi, 2009). These characteristic sequences located in motifs 3, 2 and 4, respectively (**Supplementary Figure 3**). Moreover, phosphorylation sites were predicted to be located in motifs 2 and 4, but not in motif 3. More importantly, some of phosphorylation sites, S362 for AhPP2C134/AhPP2C45, S322 for AhPP2C35/AhPP2C121, S532 for AhPP2C59 and S531 for AhPP2C150, were within the G××DN characteristic sequence. These phosphorylation sites may provide an important direction for future studies of AhPP2Cs in ABA-related signal transduction under salt stress.

Based on phylogenetic, gene structure, motif and *cis*-acting elements analysis of *AhPP2C-A*, *AhPP2C45* and *AhPP2C134* had similar structures to the reported *PP2C* genes associated with salt tolerance in rice and *Arabidopsis* and contained more *cis*-elements that related to salt tolerance. According to the salt stress effects on the expression, *AhPP2C45* and *AhPP2C134* had significant differences compared with the control. These results indicated that *AhPP2C45* and *AhPP2C134* could be candidate *PP2Cs* conferring salt tolerance. The study of *AhPP2C*s in response to salt stress may be used in conjunction with phenotypic data to screen for more salt tolerant genotypes and salt tolerant varieties, providing a crucial foundation for the next step breeding.

## 5 Conclusion

In this study, we identified 178 *AhPP2C* genes which distributed across the 20 chromosomes, with segmental duplication in 78 gene pairs. Phylogenetic tree, gene and protein structures, *cis-*elements and expression analysis indicated that *AhPP2C45* and *AhPP2C134* could be candidate *PP2Cs* conferring salt tolerance. These results provide important information for the research of salt-stress mechanism, selection of salt-resistant germplasm and breeding of salt-tolerance cultivar in peanut.

## Data availability statement

The datasets presented in this study can be found in online repositories. The names of the repository/repositories and accession number(s) can be found below: The public repository of NCBI under the biological project accession number, PRJNA560660.

## Author contributions

FL conceived the study, designed the experiments, supervised and complemented the writing. YW provided suggestions and supervision. ZW and LL produced the figures and wrote the original article. All authors discussed the results and commented on the article. All authors contributed to the article and approved the submitted version.

## Footnotes

1
https://www.peanutbase.org/peanut_genome

2
https://www.ncbi.nlm.nih.gov/cdd/

3
http://mg2c.iask.in/mg2c_v2.1/

4
https://web.expasy.org/protparam/

5
http://gsds.gao-lab.org/

6
http://bioinformatics.psb.ugent.be/webtools/plantcare/html/

7
https://npsa-prabi.ibcp.fr/cgi-bin/npsa_automat.pl?page=npsa_sopma.html

8
https://swissmodel.expasy.org/in⁃teractive

9
https://blast.ncbi.nlm.nih.gov/Blast.cgi

10
https://services.healthtech.dtu.dk/service.php?NetPhos-3.1

11
http://www.sbg.bio.ic.ac.uk/phyre2/html/page.cgi?id=index

12
http://eggnog-mapper.embl.de/

13
https://www.NCBI.nlm.nih.gov

## Supplementary material

Supplementary Figure 1 Motifs analysis of PP2Cs in Subclades AI and AII.

Supplementary Figure 2 GO and KEGG enrichment analysis of clade A *AhPP2C*s. **(A)** The enriched GO terms from MF, CC, BP classifications in *AhPP2C*s. **(B)** The enriched KEGG pathways in *AhPP2C*s.

Supplementary Figure 3 Sequence alignment of PP2Cs in subclades AI and AII.
