# Supplementary figures and images for: Genome-wide characterization of the PP2C gene family in peanut (Arachis hypogaea L.) and the identification of candidate genes involved in salinity-stress response
Source: Front Plant Sci. 2023 Jan 27;14:1093913. doi: 10.3389/fpls.2023.1093913 (PMC9911800; doi:10.3389/fpls.2023.1093913)

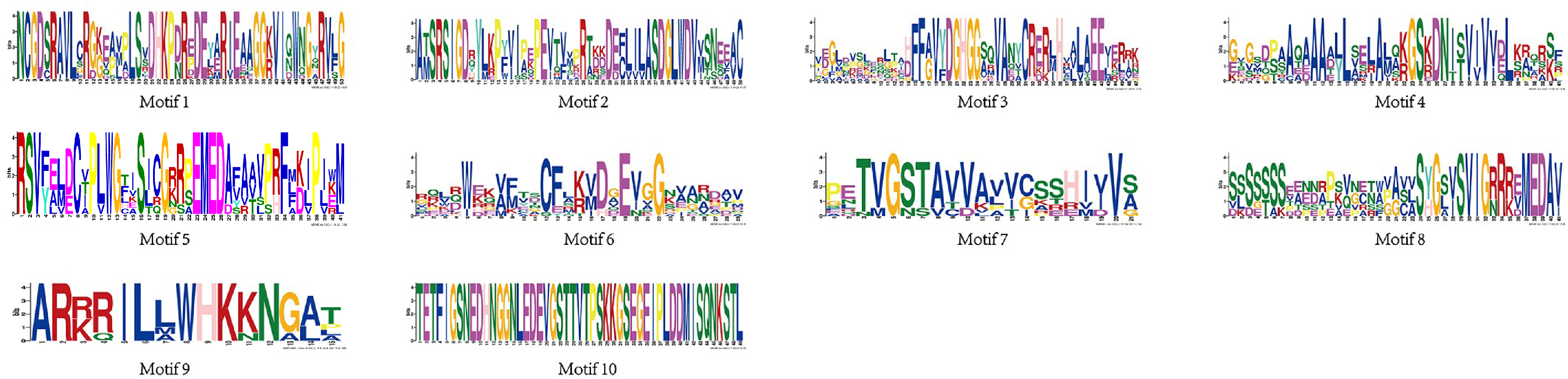

Supplement: Supplementary Figure 1 — Motifs analysis of PP2Cs in Subclades AI and AII. [file Image_1.jpeg]

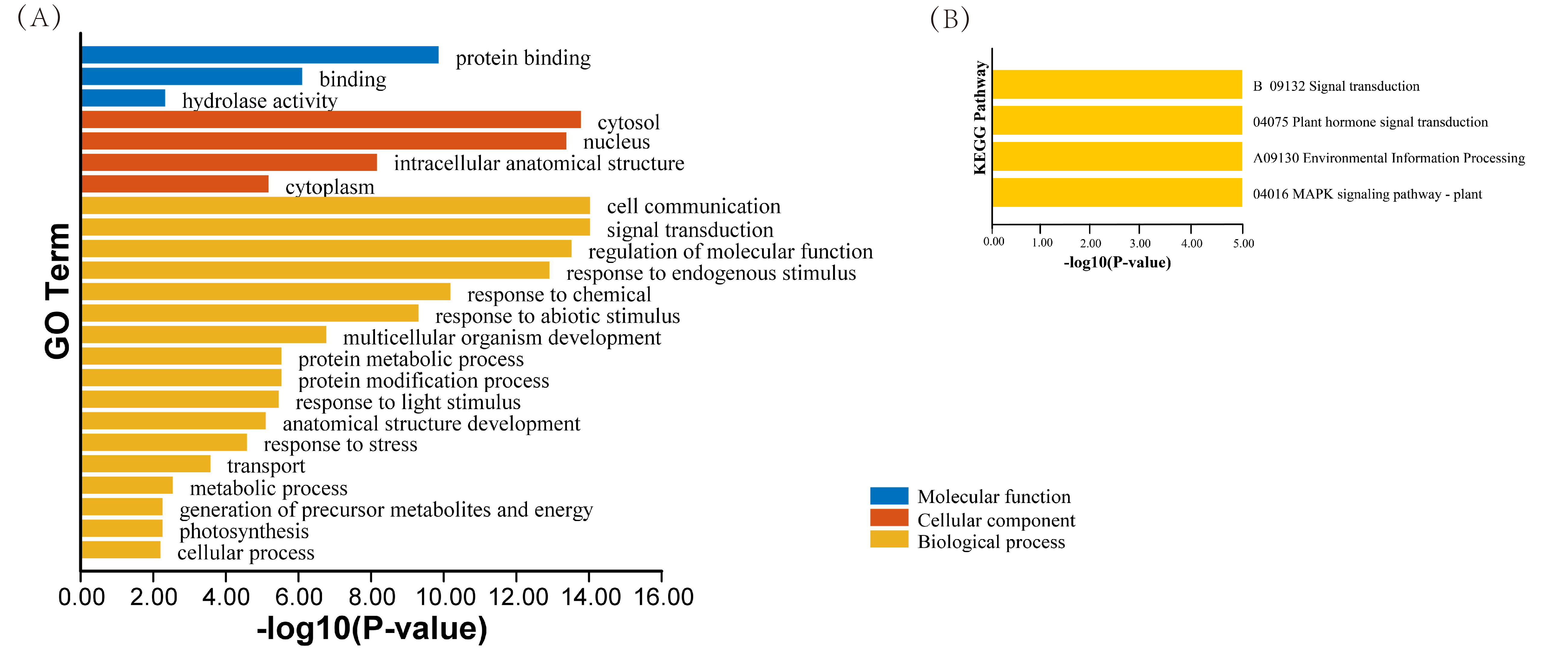

Supplement: Supplementary Figure 2 — GO and KEGG enrichment analysis of clade A AhPP2Cs. (A) The enriched GO terms from MF, CC, BP classifications in AhPP2Cs. (B) The enriched KEGG pathways in AhPP2Cs. [file Image_2.jpeg]

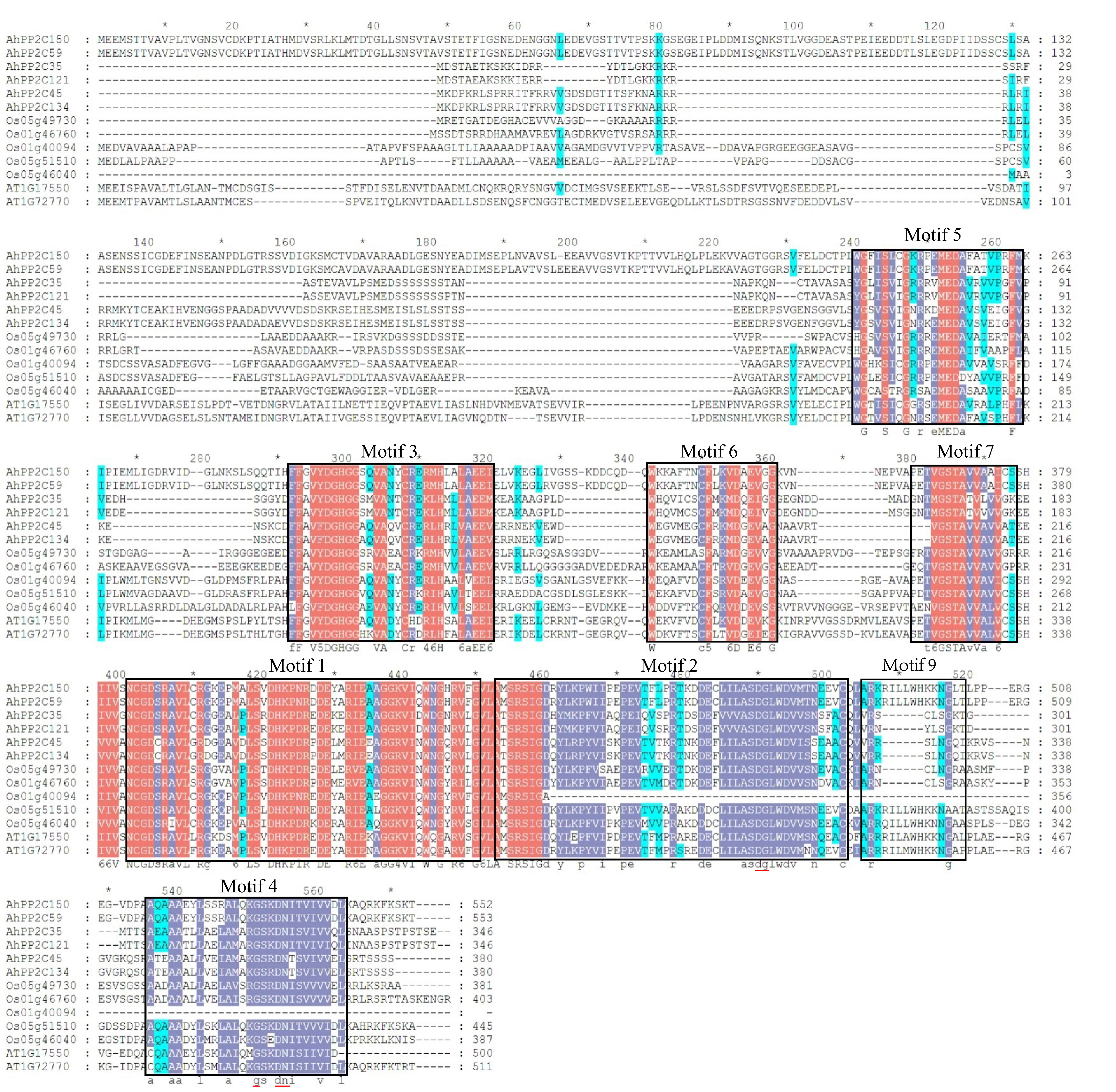

Supplement: Supplementary Figure 3 — Sequence alignment of PP2Cs in subclades AI and AII. [file Image_3.jpeg]
